# Relying on more sense for enhancing lower limb prostheses control: a review

**DOI:** 10.1186/s12984-020-00726-x

**Published:** 2020-07-17

**Authors:** Michael Tschiedel, Michael Friedrich Russold, Eugenijus Kaniusas

**Affiliations:** 1 Research Group Biomedical Sensing, TU Wien, Institute of Electrodynamics, Microwave and Circuit Engineering, Vienna, 1040 Austria; 2 Global Research, Ottobock Healthcare Products GmbH, Vienna, 1110 Austria

**Keywords:** Prosthesis control, Artificial limb, Locomotion mode estimation, Terrain, Environment, Contralateral, Systematic review

## Abstract

Modern lower limb prostheses have the capability to replace missing body parts and improve the patients’ quality of life. However, missing environmental information often makes a seamless adaptation to transitions between different forms of locomotion challenging. The aim of this review is to identify the progress made in this area over the last decade, addressing two main questions: which types of novel sensors for environmental awareness are used in lower limb prostheses, and how do they enhance device control towards more comfort and safety. A literature search was conducted on two Internet databases, PubMed and IEEE Xplore. Based on the criteria for inclusion and exclusion, 32 papers were selected for the review analysis, 18 of those are related to explicit environmental sensing and 14 to implicit environmental sensing. Characteristics were discussed with a focus on update rate and resolution as well as on computing power and energy consumption. Our analysis identified numerous state-of-the-art sensors, some of which are able to “look through” clothing or cosmetic covers. Five control categories were identified, how “next generation prostheses” could be extended. There is a clear tendency towards more upcoming object or terrain prediction concepts using all types of distance and depth-based sensors. Other advanced strategies, such as bilateral gait segmentation from unilateral sensors, could also play an important role in movement-dependent control applications. The studies demonstrated promising accuracy in well-controlled laboratory settings, but it is unclear how the systems will perform in real-world environments, both indoors and outdoors. At the moment the main limitation proves to be the necessity of having an unobstructed field of view.

## Background

The amputation of a limb is an irreversible intervention into the physiological integrity of a human being. Limb-loss is often caused by cardiovascular complications or diabetes; increasing obesity and aging population are the main contributing factors [1, 2]. Recent projections indicate that the number of major limb amputations will increase substantially [1].

Passive prostheses can replace the missing body parts to a high degree and improve patients’ independence and mobility. However, these devices lack the capability of generating power and therefore result in higher metabolic expenditure, increased stress to other joints and an asymmetric gait [3]. An unphysiological gait, especially reduced toe clearance, increases the risk of falling. Modern active powered prostheses have the capability to overcome this issue by providing net positive work required in daily activities [4]. But the question arises: do we already have the best sensor and control concepts to integrate such devices seamlessly into the patients’ lives and autonomously adapt to their needs?

**Fig. 1:**
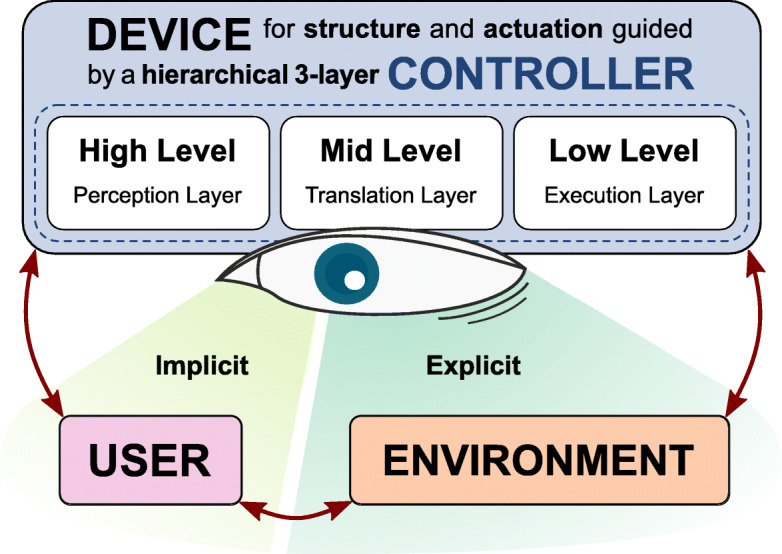
Control framework. Dynamics between a prosthetic device, a user, and his environment. The hierarchical controller estimates the patient’s intent at the high-level, translates it into device states at the mid-level and finally executes these commands at the lower level. Environmental awareness is achieved by observing the user (IES) or the environment (EES). Adapted from Tucker et al. 2015 [6]

Focusing on lower limb prostheses, many state-of-the-art devices use finite-state controllers, decomposing the gait into a series of distinct phases with a discrete set of parameters [5]. In 2015, Tucker et al. [6] conducted a comprehensive review on control strategies for lower extremity prosthetics and orthotics. The ideas by Varol et al. 2010 [7] were extended to a generalized control framework consisting of four major sub-blocks: the Controller, the Device, the User and the Environment, as depicted in Fig. 1. The *Controller* can be represented as a three level hierarchy. At the highest level, the system is responsible for correctly estimating the patient’s intent. Different terrains like level ground, stairs or ramps are related to different locomotion modes. The proper identification of transitions between different forms of locomotion is the most challenging task. The mid-level layer maps the estimated locomotion mode to the desired state outputs of the device. Finally, at a low-level, feedforward and feedback controllers minimize the error between the current state and the reference. The *Device* itself contains the mechanical and actuation structure for restoring or assisting the human functional morphology. The *User* and the device should work together in an intuitive and synergistic way, in which the device supports the patient’s motion intentions. From the perspective of the device everything else is *Environment*. Tucker categorizes the environment interaction into implicit environmental sensing and explicit environmental sensing. *Implicit Environmental Sensing* (IES) creates an understanding of the locomotion mode by measuring the state of the residual patient’s body *Explicit Environmental Sensing* (EES), on the other hand, tries to directly estimate terrain features.

In order to guarantee a safe and comfortable control, a seamless estimation of IES and EES is required. In recent years, the automotive and robotic industry have driven innovation and development mainly in the fields of TOF (time of flight) cameras, LIDAR (light detection and ranging) systems or RADAR (radio direction and ranging) solutions. This resulted in reduced prices for evaluation kits with powerful computer vision tools.

For the first time, the progress made in this area over the last ten years will be identified, focusing on the modalities of the sensors used in lower limb prostheses and on the strategies for enhancing device control. From this novel perspective, we conclude by outlining the most promising approaches and improvements that could make “next generation prostheses” more user-friendly, functional and safe.

## Methods

The selection process for this review combined three different search strategies.

Firstly, the comprehensive review from Tucker et al. in 2015 [6] was used as starting point for the snowballing approach [8], going backward from Tuckers paper by reviewing the reference list as well as going forward by identifying articles citing this publication.

Secondly, a systematic literature review, based on the PRISMA [9] guidelines was conducted. Therefore, a search string was defined for retrieving publications of interest from two different databases (i.e. IEEE Xplore and PubMed.gov). In order to find relevant articles, the first term was either “prosthe*”, “extremity” or “limb”. It was connected via a logical AND with either “radar”, “lidar”, “time-of-flight” or “depth” for focusing on dedicated sensor expressions as well as with “terrain”, “environment” or “locomotion” for more holistic synonyms. Duplicates were removed, title, abstract and full publication were screened, and the following inclusion and exclusion criteria were applied to select or reject publications:

**Criteria for inclusion:** Strategies for estimating environmental information to improve existing prosthesis control as well as all types of locomotion modes were included. Only portable prototypes were considered. The application for enhancing “prosthesis control” must be mentioned. Only articles published in “English” during the last 10 years (i.e. 2009 – 2019, final update: 12 November 2019) were included.

**Criteria for exclusion:** Systematic reviews and literature reviews, any kind of upper extremity solution, exoskeletons or orthotics-related papers were excluded. Systems based on inertial measurement units (IMU), for analyzing human motion (gait) without any link to prosthesis control are not in the focus of this review. Also not included were studies focusing only on neuromuscular or mechanical signals from the device itself or the residual ipsilateral limb. Computer vision publications without association to enhancing prosthesis control were excluded as well.

Finally, the selected publications of the outlined search strategies were used for an author cross-check. The publication lists of all referred authors retrieved from Google Scholar, ORCID or institutional and private websites were rechecked to see if individual publications meet the inclusion criteria. For example, if an earlier conference paper was discovered in the database search, but the same author had also published a journal paper covering the topic of interest, which was not caught by the first two search methods, it was also included in this review.

## Results

**Fig. 2:**
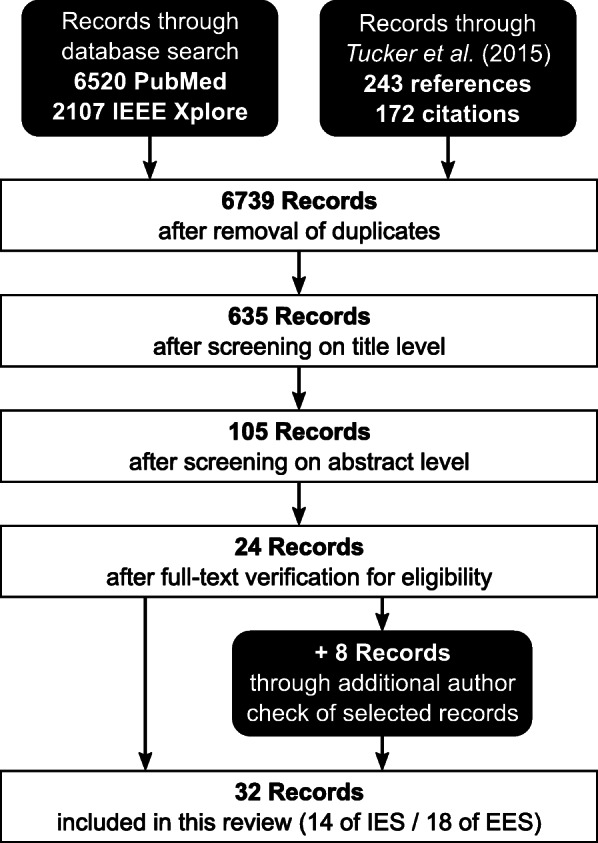
Search process. Flow diagram of database search and paper selection based on inclusion and exclusion criteria throughout the different phases of the literature review process

An overview of the selection process is shown in the flow diagram in Fig. 2. Twenty four out of the 6739 articles identified with the search strategies met the inclusion criteria. Another 8 were added through the final author cross-check, resulting in 32 publications included in this review.

### Overview

The retrieved 32 publications were categorized by the two types of environment. The majority (18) of those are related to EES, the remaining (14) to IES. Table 1 provides a summary of all included records. The main characteristics of the publications are structured in the following columns:

**Table 1:** Overview of records reviewed

**Study**	**Type / Group**	**Sensor selection**	**Sensor placement**	**Concept description**
Vallery et al.	IES / 1	2 x angle & angular	**C**: hip & knee	Mapping function for control of knee prototype
(**P**, 2011) [10]		velocity sensors		with estimated contralateral limb motion data.
Bernal-Torres et al.	IES / 1	1 x IMU	**C**: thigh	Active biomimic polycentric knee prototype with
(**H**, 2018) [11, 12]				contralateral echo-control strategy.
Su et al.	IES / 1	3 x IMUs	**C**: thigh, shank &	Intent recognition system based on
(**P**, 2019)[13]			ankle	convolutional neural network classification.
CYBERLEGs	IES / 1	2 x pressure insoles	**B**: shoes inlays	Finite-state control of a powered ankle-knee
project series ^1^		7 x IMUs	**B**: thighs, shanks,	coupled prototype using whole-body aware
(**P**, 2017) [15–18]			feet & 1 x trunk	noninvasive, distributed wireless sensor control.
Hu et at.	IES / 2	4 x IMUs	**B**: thighs & shank	Classification error reduction through fusion of
(**P**, 2018) [19–21]		4 x GONIOs	**B**: knee & ankle	bilateral lower-limb neuromechanical signals,
Extended by:		14 x EMGs	**B**: leg muscles	providing feasibility & benchmark datasets.
Krausz et al.	EES / 2	1 x IMU	On the waist in	Adding vision features to the prior
(**H**, 2019) [22]		1 x depth camera	a belt construction	concept improving the classification.
Hu et al.	IES / 3	1 x IMU	**I**: thigh	Bilateral gait segmentation from ipsilateral depth
(**H**, 2018)[23]		1 x depth camera		sensor with the contralateral leg in field of view.
Zhang et al.	IES / 3	1 x depth camera	On the waist	Depth signal from legs as input to an
(**H**, 2018) [25]			with tilt angle	oscillator-based gait phase estimator.
Scandaroli et al.	EES / 4	2 x gyroscopes	Built into a	Infrared distance sensor setup for estimation
(**T**, 2010) [27]		4 x infrared sensors	foot prototype	of foot orientation with respect to ground.
Ishikawa et al.	EES / 4	2 x infrared sensors	Left & right on	Infrared distance sensor setup for estimation
(**H**, 2018) [28]		1 x IMU	one normal shoe	of foot clearance with respect to ground.
Kleiner et al.	EES / 5	1 x motion tracking	**I**: between artificial	Concept and prototype of a foresighted
(**T**, 2011) [29]		1 x laser scanner	ankle & knee joint	control system using a 2D laser scanner.
Huang’s group ^2^	EES / 5	1 x IMU	**I**: lateral side	Terrain recognition based on laser distance,
(**P**, 2016) [30–33]		1 x laser sensor	of the trunk	motion estimation and geometric constrains.
Carvalho et al.	EES / 5	1 x laser sensor	On the waist	Terrain recognition based on laser distance
(**H**, 2019) [36]			with 45° tilt angle	information and geometric constrains.
Sahoo et al.	EES / 5	3/4 x range sensors	**I**: On the shank &	Array of distance sensors for geometry-based
(**H**, 2019) [37]		1 x force resistor	on the heel of the foot	obstacle recognition in front of the user.
Varol et al. and	EES / 5	1 x depth camera	**I**: shank	Intent recognition framework using a single
Massalin et al.				depth camera and a cubic kernel support
(**H**, 2018) [38, 39]				vector machine for real-time classification.
Laschowski et al.	EES / 5	1 x color camera	Wearable	Terrain identification based on color images
(**H**, 2019) [40]			chest-mounting	and deep convolutional network classification.
Yan et al.	EES / 5	1 x depth camera	On the trunk	Locomotion mode estimation based on depth
(**H**, 2018) [41]			in 1.06m height	feature extraction and finite-state classification.
Diaz et al.	EES / 5	1 x IMU	**I**: foot & shin	Terrain context identification and inclination
(**H**, 2018) [43]		1 x color camera		estimation based on color image classification.
Krausz et al.	EES / 5	1 x depth camera	Fixed in 1.5m height	Stair segmentation strategy from depth
(**H**, 2015) [45]		1 x accelerometer	with -50° tilt angle	sensing information of the environment.
Kleiner et al.	EES / 5	1 x IMU	**I**: thigh	Stair detection algorithm through fusion of
(**P**, 2018) [46]		1 x radar sensor		motion trajectory and radar distance data.
Zhang et al.	EES / 5	1 x IMU	**I**: knee lateral	Environmental feature extraction based on
(**P**, 2019) [47, 48]		1 x depth camera		neural network depth scene classification.

^1^Publications through CYBERLEG: Amrozic et al. [15, 16], Gorsic et al. [17] and through CYBERLEG++: Parri et al. [18]

^2^Research group from Huang: F. Zhang et al. [30], X. Zhang et al. [31], Wang et at. [32] and Liu et al. [33]

**Study:** In this column, the first author as well as the publication year and the reference are mentioned. If more than one reference is given, the year indicates the most recent publication. If research groups have performed tests with amputees, this is indicated by (P). If they have evaluated their system only with healthy subjects, it is marked with (H). (T) implies that it is a theoretical concept, eventually tested in an experimental setup, but not tried in interaction with human beings.

**Type / Group:***Type* serves as an indicator, to show if the record is more related to implicit or to explicit environmental sensing. *Group* assigns each publication a particular control strategy out of five main categories retrieved within this review – a detailed explanation is given in “Discussion” section. The overview table is sorted by this column.

**Sensor selection:** This column describes the type of sensors used in the study.

**Sensor placement:** This column gives an overview, where the respective sensors are placed on the human body. The lower part of the human body is segmented into foot, shank, thigh and trunk. The connecting joints are ankle, knee and hip. Bilateral (B) means “on both sides of the body”. Ipsilateral (I) means “located on the same side of the body part” or respectively on the same side as the device. The opposite is contralateral (C) which signifies “located on the opposite part”.

**Concept description:** This field shortly summarizes how environmental information is used for enhancing prosthesis control in each record.

**Fig. 3:**
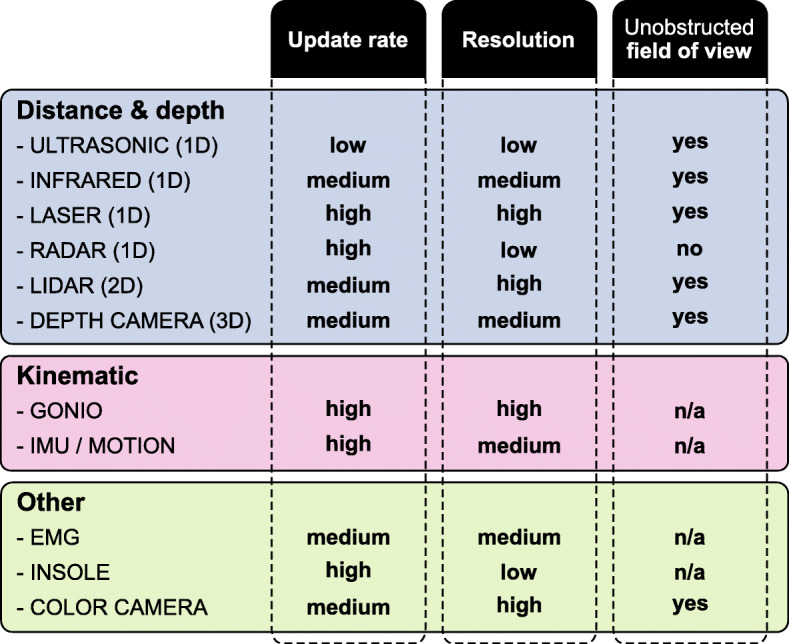
Sensor comparison. Different sensors used within the retrieved publications were divided into the three categories: *Distance & depth*, *Kinematic* and *Other*. *Update rate* describes the number of measurements per second. The rating scale: (low), (medium) and (high) is used instead of absolute values, representing a scale from approximately 10 Hz up to 100 Hz for real-time applications. The smallest change that can still be detected by a sensor is its *Resolution*. The rating scale: (low), (medium) and (high) is used instead of absolute values, representing a scale from several centimeters down to the millimeter range. *Unobstructed field of view* indicates whether the sensor functionality does or does not require an unobstructed field of view: (yes/no). If its not applicable, this is indicated by: (n/a)

Within the publications, 11 different types of sensors were used. It was possible to divide those sensors into three categories, as shown in Fig. 3. In particular, *Distance & depth* differentiating sensors based on ultrasonic or electromagnetic waves with different frequencies and *Kinematic* grouping sensors for measuring the motion of bodies. EMG electrodes, pressure insoles and color cameras were summarized into *Other*. Information was extracted from the reviewed publications itself and, if missing, completed with the help of the manufacture’s datasheet. The main characteristics are the sensor update rate, resolution and the need for an unobstructed field of view.

### Implicit environmental sensing

Vallery et al. [10] presented a complementary limb motion estimation strategy. In this application, a linear mapping function outputs the state of the missing limb dependent on the state of the residual sound side. The angle and angular velocity is measured by sensors attached to the contralateral hip and knee. So far, only results from one above-knee amputee were presented. The patient was almost able to achieve a physiological gait pattern. However, detailed technical information was not given.

Instead of using a monocentric knee prototype, Bernal-Torres et al. [11, 12] proposed a concept of an active polycentric knee prosthesis using the echo-control schema. An inertial measurement unit fixated on the contralateral thigh estimates the trajectory of the unimpaired knee. The average tilt angle error between the polycentric knee prototype, mounted in a test workbench, and the anatomical lower limb of one non-impaired subject was about 2°.

Three IMUs on the contralateral thigh, shank and ankle for locomotion intent recognition, were used by Su et al. [13]. The sensor data was taken as input into a convolutional neural network, a class of computational processing systems heavily inspired by biological neural networks. It “learns” to perform tasks using self-optimizing weights and biases throughout example-based learning. Filters are used to extract hierarchical patterns in date, which makes them particularly interesting for (image) recognition systems [14]. Ten able-bodied subjects and one above-knee amputee participated in the study. Different strategies for user-independent and user-dependent classification with varying amount of test and training data were analyzed. The highest accuracy was reported with 94.2% for the able-bodied and 89.2% for the amputees, classifying five types of terrains and eight transitions between them.

As part of the CYBERLEGs project Ambrozic et al. [15, 16] and Gorsic et al. [17] used the *α*-prototype prosthesis (actuated ankle and a passive knee) with a “whole-body aware” control approach. The user intention was measured through seven wireless IMUs, attached bilaterally to the feet, shanks and thighs and one on the trunk. Additionally, two pressure insoles measured the ground force and the center of pressure. The control scheme combined the intent detection from the body-worn sensors and the prosthesis control into one state machine with unified states and transitions based on the analysis of gait in healthy subjects. The overall intent recognition for three unilateral transfemoral amputees was accurate in 85.2% of the cases for level-ground walking. Parri et al. [18] used a similar wearable sensory concept and the advanced *β*-prototype throughout the CYBERLEGs Plus project. In this study, four unilateral transfemoral amputees participated in the study with different activities. 100% accuracy was reported in treadmill walking, even at a low walking speed. The lowest score was achieved for the sit-to-stand task at 94.8%.

A systemic analysis of different signals from the contralateral side for predicting the locomotion mode was done by Hu et al. [19–21]. Ten healthy subjects participated in the study generating a public available benchmark dataset of lower limb joint kinematic and electromyography (EMG) data, simultaneously recorded with wearable sensors. Electrogoniometers (GONIO) were used to record joint kinematic signals of knee and ankle of both legs. IMUs were placed bilaterally on the subject’s thigh and shank. Bipolar surface EMG electrodes were placed on seven muscles in each leg. They analyzed different combinations of sensors and algorithms. It was shown that only one additional contralateral sensor could significantly reduce intent recognition error rates. Finally, an offline analysis of one above-knee amputee walking with a powered leg prosthesis was presented. Placing two additional IMUs on the contralateral thigh and shank could reduce overall, steady-state and transitional error rates by more than 60%, compared to ipsilateral sensor placement as baseline. Parallel to this, Krausz et al. [22] extended the system by an IMU and a single depth camera. These sensors were worn on a belt-like construction with the environment in front of the subject in the field of view. The IMU was used to transform the vision information into a global reference system. Each frame was segmented into a grid of regions of interest before extracting three types of vision-based features: distance and orientation, motion information and the projected shape of the terrain on them. The influence of each sensor modality was analyzed, reporting that adding “vision information” increases the repeatability and, at the same time, reduces the variability across subjects and locomotion modes.

Despite the positive outcomes, the additional instrumentation of the non-prosthetic side is not really practical and comfortable for amputees. Hu et al. [23] extended their ideas and presented a novel method for bilateral gait segmentation using only unilaterally worn sensors. A single IMU and a depth camera were placed on one thigh to detect bilateral gait events. RANSAC [24], an iterative method to estimate a model in a set of data containing outliers, was used to identify the ground plane in the depth points. Vision filtering and grouping methods were applied to correctly estimate the shank angle of the contralateral leg. IMU data and sound side features were fused for intent classification. The system was tested with one healthy subject showing that it is possible to detect bilateral gait events even from unilaterally worn sensors.

Zhang et al. [25] conducted a study, in which both legs were within the sensor’s field of view. A depth camera was mounted on the waist looking forward with such a tilt angle, that the toes are just not captured when the person is standing still. A movement led to a periodic variation of depth values. This signal was then used as input into an earlier published concept of an adaptive oscillator gait phase detector [26], a method for extracting features and synchronizing to periodic signals. Four able-bodied subjects participated in a level ground walking study, reporting a maximum estimation error of 0.3 rad between the estimated gait phase and the reference gait phase calculated out of two consecutive steps.

### Explicit environmental sensing

Foot clearance is an important gait parameter and serves as an indicator for gait quality and safety. Scandaroli et al. [27] presented a prototype of a prosthetic foot equipped with two gyroscopes and four infrared distance measuring elements. The concept was to estimate foot orientation with respect to the ground. So far, only test-bench results estimating the inclination and height of the foot above the ground have been presented. Ishikawa and Murakami [28] equipped a normal shoe with two infrared distance sensors and one IMU. The data gathered from one healthy subject walking in five different terrains was analyzed. The waveform of the sensor signal was reported to be unique for different locomotion modes – a dominant double peak was the characteristic of leveled walking, but detailed technical information was not given.

Already in 2011, Kleiner et al. [29] published a concept of a foresighted control for a foot prosthesis. An optical measuring system consisting of a laser scanner and an inertial navigation system was mounted between the ankle and the knee on the side of the prosthesis. The two-dimensional (2D) depth data from the laser scanner was combined with the motion information from the inertial system to create a three-dimensional (3D) representation of the environment. The idea was to use computer vision methods in order to detect objects like stairs, or ramps in the environment. So far, only “images” from a single indoor experiment were presented without any technical details.

Instead of using a 2D laser scanner, the research group from Huang [30–33] used a single laser distance meter and one IMU for terrain recognition. They extended the concept of a locomotion mode recognition system based on neuromuscular EMG signals from the residual limb and mechanical load information on the device [34, 35]. The additional sensors were mounted laterally on the trunk of the prosthetic side. A decision tree classified the terrain in front of the user into five different categories depending on thresholds and geometric constrains. The system was tested on six able-bodied subjects and one above-knee amputee. It identified the new terrain 500 ms before executing locomotion mode transition with an accuracy of 98%.

A concept without an IMU was introduced by Carvalho et al. [36]. The information from an infrared laser mounted on the user’s waist was classified with a three-layer decision tree with heuristic rules. Tested on 10 able-bodied subjects, the classification accuracy for eight locomotion mode transitions was above 80%, achieving 100% success for identifying the transition from ramp descent or stair descent into level ground.

An array of distance sensors was used by Sahoo et al. [37]. In this study, a prototype with either four ultrasonic distance sensors or three laser distance sensors was mounted on the shank of the participant. Reliable measurements were always taken during the stance phase triggered by a force resistor attached at the heel of the foot. The distance signals were used to classify four types of terrains ahead of the user. Two classification approaches, such as quadratic discriminant analysis and rule-based system, were explored with two able-bodied subjects. The ultrasonic sensors achieved an accuracy above 97%, however the range within obstacles were detected was less than 50 cm leading to the risk to “miss a transition” if the step length of the subject was greater than the detection range. In comparison, the laser distance sensors increased this range up to 100 cm. By taking the most frequent prediction class within a single step, the system identified the new terrain 650 ms before executing locomotion mode transition with an accuracy of above 98%.

Varol et al. [38] and Massalin et al. [39] attempted to detect five different locomotion modes with a depth sensor. In this application, a single depth camera was mounted unilaterally on the shank with a 45° tilt angle to the ground plane. In order to embed motion information, so called “depth difference images” were calculated. This was done through pixelwise subtraction of the preceding depth frame. Twelve healthy subjects participated in the study. Data of eight subjects was used to train different variations of support vector machine classifiers, a supervised learning algorithm that sorts data into predefined categories. The highest reported accuracy of 94.1% was achieved with a cubic kernel and no dimension reduction classifier on the test-data of the remaining four subjects. The averaged computation time was reported with 14 ms.

Three different types of terrains were classified with an overall accuracy of 94.85% in the study from Laschowski et al. [40]. A chest-mounted color camera with the environment in front of the subject in its field of view was used for data acquisition. One able-bodied subject walked around, collecting over two million sample images. Around 34,000 of them were individually labeled to train the 10-layer deep convolutional neural network used for classification.

Yan et al. [41] presented a depth image-based locomotion recognition approach that does not require any pre-training. A depth camera mounted on the waist in a height of 1.06 m, having the terrain and a small portion of the user’s feet in its field of view, is used in this setup. Depth images were segmented into 12 blocks and locally averaged. A finite-state machine with predefined thresholds is then used to classify between four locomotion modes. Additionally, stair edges were detected by using a Hough Line Transform [42], a feature extraction technique to find a certain class of shapes by a voting procedure. Nine healthy subjects participated in the study. The accuracy for steady state locomotion tasks was reported as 100%. However, correctly detecting the transitions was challenging. Nevertheless, 82.4% of the terrain changes could be detected before executing a locomotion mode transition. In this study, there was no real-time evaluation performed, although the computation time was only 5 ms.

Rather than classifying the locomotion mode of the user, Diaz et al. [43] proposed a concept of estimating the soil properties as well as the surface inclination in front of the user’s leg. In this application, a normal color camera was mounted on the shin and an IMU on the top of the foot of an able-bodied subject. Comparable images were always taken during the stance phase of the gait cycle. The images were classified with the Bag of Words method [44], analyzing the input against a predefined bag of local image features. The classifier was able to identify 6 types of terrains (asphalt, carpet, cobblestone, grass, mulch and tile) with an averaged accuracy of 86%. The prediction of the terrain inclination in front of the leg was accurate to 0.76° compared with a reference.

Krausz et al. [45] presented a method for estimating stair parameters with a depth vision system in 2015. The proposed algorithm used prior knowledge of a basic stair structure and input of a single three-axis accelerometer. One able-bodied subject held the camera in 1.5 m in height with a -50° tilt angle from horizontal and walked through a hallway entering into a stairwell. This online test resulted in 98.8% accuracy for classification if they were either “approaching” stairs or “not approaching”.

The first and only group using a RADAR sensor for stair detection was Kleiner et al. [46] in 2018. In this application, a radar distance sensor and an IMU were mounted on the thigh of the prosthetic device. Fusing both signals created a 2D image of the sagittal plane in its virtual field of view. For objects in a range up to 5 meters in front of the device, a mean accuracy of 1.5 ±0.8 cm was reported. The mean accuracy for height estimation lies within 0.34 ±0.67 cm.

The most advanced environmental feature recognition system was presented from Zhang et al. [47]. In this application, five different environments could be distinguished. A depth camera and an IMU mounted ipsilaterally on the knee joint were combined to transform the captured scene into world coordinates. The 3D scene was reduced to a 2D binary image, which reduced total computing time remarkably to only 23 ms. A deep convolutional neural network was used to classify the type of input scene. Finally, after classifying the type of terrain, basic computer vision methods were used to estimate features such as the slope angle of a ramp or the height and width of stairs. The proposed system was evaluated using data from simulation, indoor and outdoor experiments. Six able-bodied subjects and three above-knee amputees participated in the study. Data from the simulation and one healthy subject was used to train the network, the remaining data was used for validation only. The classification accuracy for amputees was reported with 99.3% for indoor and 98.5% for outdoor experiments, predicting the terrain change at least 0.6 s before switching the locomotion mode. The latest publication from Zhang et al. [48] considered the credibility of decisions and the relationship between states for improving the classification even further.

## Discussion

Commercially available lower limb prostheses use mainly device-embedded sensors to “(re)act” to the patient’s intent. However, due to the lack of contextual (environmental) information, misclassifications can result in stumbling or even falling down. The present review describes the progress made over the last decade towards more “foresighted” prosthetic systems. From this novel perspective, a “control strategy landscaping” was derived, how environmental information can enhance “next generation prostheses”.

### Control strategy landscape

**Fig. 4:**
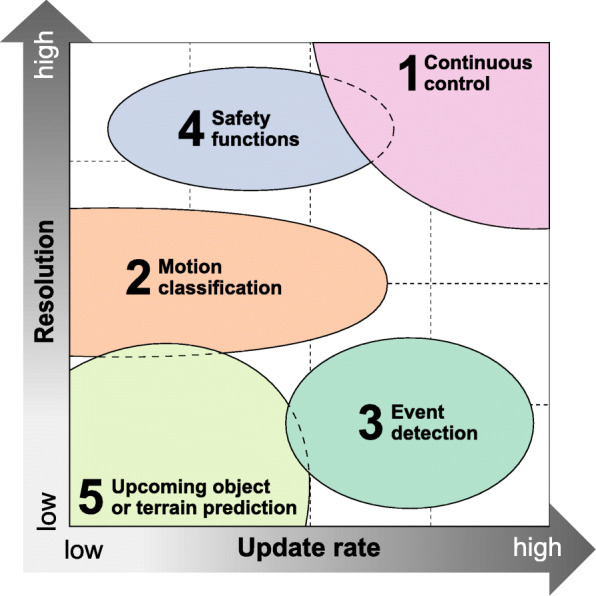
Control landscape. Control strategy landscape overview based on required resolution and update rate of the underlying sensor modality

New concepts for environmental sensing in “next generation prostheses” can be distinguished according to how they improve existing control systems. As depicted in Fig. 4, enhanced control strategies can be separated into five groups, namely continuous control (1), motion classification (2), event detection (3), safety functions (4), and upcoming object or terrain prediction (5). The required update rate and resolution is a criterion to select the best sensor modalities for each of them. All retrieved publications were assigned into one of these main groups, dedicated in column 2 of Table 1.

*Continuous control* systems measure and adjust in real time. Hence, sensors with high update rates as well as high resolutions are needed. In accordance with three-level controller hierarchy framework from Tucker, input is processed directly at mid-level layer to control the prosthetic device state in real time. In this category, only IES-based residual patient’s body estimation strategies were investigated. Typically, kinematic sensors like IMUs or GONIOs are used. Using contralateral leg information seems to be a common concept [10–13]. However, simple echo-control strategies caused errors, especially at the beginning and the end of an activity, when the limbs are not required to “echo” each other. Placing sensors not only on the contralateral leg, but rather measuring the entire (lower) body, expands the possibilities. These concepts [15–18] can control in real-time throughout numerous locomotion modes and transitions between them. However, for all the presented “body aware” concepts, it was necessary to mount additional sensors on the patient’s body, which reduced usability.

Not controlling, but rather classifying is meant by *motion classification*. Signals measured from distributed sensors are used to perceive the patient’s intent at the high-level layer of the controller hierarchy framework better. Therefore, sensor update rates as well as resolutions are typically lower. Unsurprisingly, fusing whole body kinematic signals lead to higher intent recognition rates, as was proven systematically [19–22]. Remarkably, however, it could be shown that an additional sensor on the unimpaired sound side can reduce misclassifications. Instead of mounting and calibrating a large number of additional sensors to the patient’s body, a single contralateral sensor would be sufficient to enhance device control. As depicted in Fig. 4, motion classification also overlaps with the group of upcoming object or terrain prediction. Although the focus of this section here lies mainly on the correct intent recognition rather than on predicting the upcoming terrain directly, these two are strongly related by nature – obstacles and terrain features are in this sense the “boundary conditions” for all movements.

Strategies extracting specific “events” from the human gait cycle are combined into *event detection*. The most prominent are the “heel strike”, initiating the stance phase, and the “toe off”, responsible for the beginning of the swing phase [49]. Normally, this information is used at the mid-level control layer to trigger movement-dependent actions. Therefore, the required sensor update rate is typically higher than for motion classification, however, the resolution can be lower. For example, pressure insoles are typically used for measuring ground contacts. While timing is very important, it can often be sufficient to differentiate, whether the foot is, or is not, in contact with the ground. A continuous gait phase estimation was carried out on the basis of a gait-related depth signal captured from a waist-worn depth camera [25]. The approach from Hu et al. [23] seems to be more practical, where a unilaterally mounted depth camera detected bilateral gait events. However, the computation time of more than 1 s prevented any real-time (online) application.

*Safety functions* summarize all concepts which contribute to the safety of a prosthetic device. In terms of environmental sensing, reliable prevention of stumbling and falling in unexpected terrains is still an open question. A study with able-bodied individuals found that an unrecognized object with a height of 1 cm can lead to stumbling, concluding that foot clearance is an important parameter to prevent falling [50]. For measuring this parameter, infrared distance sensors were used in two publications [27, 28], but not evaluated in a prosthetic setup. In general, the resolution of the sensor modality has to be high enough to correctly detect small barriers, ideally with a high update rate to “(re)act” in real time. While high resolution is mandatory, update rates can also be lower. For instance, a leg-mounted depth or color camera, capturing a single image during the mid-stance phase when the leg is almost vertical, could minimize stumbling risks during the next step. It is also conceivable that two distance sensors, one more toe-related and the other more heel-related, in combination with an IMU, could estimate the correct ground inclination. This could be especially interesting for the further development of active ankle devices, but it was not evaluated in any of the reviewed publications.

Half of all the reviewed studies deal with *upcoming object or terrain prediction* concepts. The underlying principle of all these publications is to observe the front environment of the user, interpret the input and then provide a probability for mode switching. This is mainly, due to the fact that the correct detection of locomotion mode transition between different terrains, e.g. switching from level-ground walking to stair ascent mode, is often challenging and unintuitive. For instance, in commercially available products, the user must switch between locomotion modes with substantially different characteristics by carrying out a predefined “special movement”. This action triggers the transition by using only sensors embedded into the prosthesis [51]. In accordance with the controller framework by Tucker, upcoming object or terrain prediction strategies provide input for the high level of the controller. Earlier listed safety functions on contrast, interact mainly at the low-level layer, increasing patient safety.

Huang et al. [35] coined the term “critical timing” for switching mode, neither interrupting the transition nor disturbing the balance. Concepts using 1D laser distance meter [30–33, 36, 37] combine the sensory input with geometric constrains to estimate upcoming terrain features early enough and accurately. Although one group used a RADAR sensor as an input device [46], the feature of “looking through” materials was not evaluated in this study.

Explicit object recognition is another approach for classifying upcoming barriers directly. The invention of low-cost high-resolution depth and color cameras opened up an entirely new field of vision-based object recognition [52]. Especially for autonomous robots, the capability to detect and classify objects correctly is critical. For example, a self-driving wheel-chair was able to successfully navigate through the hallways of a hospital [53]. However, in terms of lower limb device control, the final decision “where to go” or “what to do” is made by the user anyway. Therefore, the sensor raw data are usually pre-processed to obtain probabilities for possible terrain changes.

Depth cameras [38, 39, 41, 45, 47, 48] were used to differentiate between a limited number of terrains and whether the user is approaching stairs. The approach of soil property estimation based on a color camera [43] was also evaluated, but not in a prosthetic setup. Nevertheless, all vision-based systems are computationally intensive and require an unobstructed field of view. Concepts using pre-trained classifiers [38–40, 43, 45, 47, 48] achieved higher accuracies compared to finite-state machines without any training [30–33, 36, 37, 41]. However, pre-trained classifier performance depends strongly on the size of training data. Moreover, predefined step sequences during the acquisition of training data can lead to an undesired bias – the system is trained with specific parameters, but in real life step sequence and walking speed are unpredictable.

In general, it is very difficult to compare the different approaches, as they use non-standardized test procedures. In summary, the research studies reported accuracies ranging from 82% to 99%. Assuming 100 locomotion mode changes per day and expecting only every tenth misclassification to cause a fall, there would still be 3 to 54 serious tumbles per month, which does not seem very promising.

### Sensor modalities

The 32 publications reviewed use 11 types of sensors, as depicted in Fig. 3. Kinematic sensors are widely used – 24 out of 32 publications, use IMUs for motion estimation. Even all five commercially available microprocessor-controlled prosthetic knees, reviewed by Fluit et al. [5], use a shank IMU as sensory input. However, the integration of IMU information tends to suffer from accumulating errors, which can lead to drifts. Alternatively, GONIOs are accurate and reliable, but they usually limit the degree of freedom of complex human joints.

Distance sensors usually use the principle of time of flight, measuring the round trip time between emitting and receiving back a specific pulse. Ultrasonic sensors are based on mechanical (acoustic) waves. The propagation speed for these sensors is limited by the speed of sound, which results in a round trip time of approximately 6 ms for an object at 1 m distance. Thus, nature limits the update rate of ultrasonic sensors. Nevertheless, they are common for close proximity applications, as they are able to detect even transparent materials like glass. By using electromagnetic waves, the round trip time is usually negligible, because the speed of light is substantially higher. The update rate of these sensors is, therefore, limited only by the processing rate of the internal hardware. Infrared sensors emit light below the visible light range. Instead, laser sensors have operating frequencies in the visible range (red or green light) or above (invisible ultraviolet range). Update rate and resolution are normally lower for ultrasonic sensors than they are for lasers. LIDAR sensors combine laser distance meters with a complex mechanical mirror system to generate high-resolution 2D or even 3D scans. However, shocks or vibrations can disrupt the moving parts in such devices. Historically seen, these sensors used to be very expensive, whereas nowadays industries have shifted to develop low-cost solid-state LIDARs for a broad application.

Depth perception refers to the ability to estimate the surrounding world in 3D – nature (human eyes) has perfected this over millions of years. Historically, color cameras, working on passive light sensors, were combined to stereo vision systems to extract depth information from well-known digital images. Performance depends primarily on the underlying stereo correspondence algorithms (depth calculation process), which tries to match pixels of the two individual images. Today, depth cameras based on the time-of-flight principle have become increasingly more available. Similar to TOF sensors, an artificial light impulse is emitted, while the reflection is simultaneously captured by multiple sensitive elements. This generates a full 3D perception at once with resolutions up to 640 x 480 pixels, small enough to be implemented into a smartphone [54]. The resolution of depth cameras, for time of flight as well as for stereo vision based concepts, is in the range of 1% of distance with update rates varying from 5 to 60 (depth) frames per second. Nevertheless, all concepts based on infrared, visible or even ultraviolet light are limited by their explicit need of an unobstructed field of view. The additional sensors must be worn over any type of prosthetic cosmetic or clothing and can, therefore, not be integrated directly into the device. RADAR technology, however, is not limited by this factor. Although radar technology was already discovered in the late 19th century, only recent developments have made new sensors with operating frequencies up to 100 GHz available. While RADAR sensors have much lower resolutions compared to optical or depth sensors, they can still operate in harsh outdoor conditions. To which degree an object is detectable, is expressed in its cross-section, a property of the target’s reflectivity [55]. Due to the high-frequency range, certain materials, such as fabrics or plastics, typically appear transparent. On one hand, this is a good feature of RADAR sensors, as it allows an implementation directly into the prosthetic device making it possible to “look-through” clothes or cosmetics (cover of an artificial limb to appear lifelike). However, on the other hand, objects or barriers made out of these materials remain undetected. Nowadays, there is a shift towards the development of super near-field RADAR sensors which are able to detect human extremities and gestures for a broad application. For example, Google’s radar-based gesture sensing technology (Project Soli) is implemented in their Pixel 4 smartphone allowing a touchless interaction [56].

For the sake of completeness, surface EMG electrodes as well as force resistive insoles are mentioned, although they are not really “environmental sensors”. Conceptually, both type of sensors require a direct contact with the body. Surface EMG electrodes with the skin to register muscle activity and insols with the foot to detect ground contact. The unobstructed field of view, mandatory for all types of cameras, has no influence on the application here. Nevertheless, especially for event detection, insoles can provide valuable information, but the sensors need to be worn either ipsilaterally, contralaterally or bilaterally in the user’s shoe. EMG information is commonly used for real-time hand prosthesis control [57], but barely in lower limb devices, since movement artifact and baseline noise contamination is more prominent there.

### Computing power & energy consumption

Nowadays, commercial prostheses have very limited *computing power*. Typically, finite-state machines with heuristic rule-based approaches are used for intent recognition and device control. In contrast, most of the reviewed studies, carried out data acquisition and analysis on powerful computers with clock rates of 3 GHz or above and memory sizes up to 32 GB. The available computing power also influences the calculation time for interpreting or extracting usable information from the complex sensory input. The delay from measuring until adapting needs to be short enough to guarantee a safe and comfortable device operation. Typically, update rates for real-time prosthetic device applications are in the range of 100 Hz [5]. Therefore, the embedded system architecture of prosthetic devices needs to be modified significantly if real-time on-board processing should be enabled. For example, Intel’s Myriad 2 is optimized for vision processing in mobile applications within 0.5 W of power envelope [58] and could also be used in lower limb prostheses.

The *energy consumption* of advanced sensors also needs to be considered, when designing and developing new systems. The power consumption of Softkinetic’s DS 325 depth camera used in [38, 39] is below 2.5 W [59]. The pmdtechnologies’s CamBoard pico flexx used in [22, 23, 47] is below 0.3 W [60]. The radar module used in [46] has a total power consumption of 5 W [61], with almost identical geometric dimensions to those of depth sensors. Ongoing development will thus reduce energy consumption of sensors and processors even further. In contrast, research and commercial active (powered) knee prostheses use actuators consuming up to 200 W [4]. Therefore, the energy consumption of additional sensors cannot be regarded as the main reasons for exclusion.

## Conclusions

In this review, we summarized both implicit and explicit approaches of environmental sensing. For this purpose, a systematic literature review as well as a snowballing analysis of the survey from Tucker et al. [6] was performed. From our novel perspective, five broad control strategies were identified, how environmental information can make “next generation prostheses” more user-friendly, functional and safe.

There is a clear trend towards more upcoming object or terrain prediction concepts, providing switching probabilities between different locomotion modes. In summary, the research studies reported accuracies ranging from 82% to 99% in well-controlled laboratory settings, but it is unclear how the systems will perform in realistic environments, both indoor and outdoor. It was also shown that implicit environmental sensing strategies in particular can significantly improve control. Furthermore, information about the contralateral leg can play a crucial role in movement-dependent control applications.

Throughout the 32 reviewed publications, 11 types of sensors were used. Technology differences were discussed, and aspects of computing power and energy consumption mentioned. The update rate and resolution were found to be essential criteria to determine a suitable control category. Distance sensors and depth cameras are widely used, but they are limited by an unobstructed field of view. Moreover, the latter also requires higher computing power for calculating interpretable features from the (complex) raw inputs. In almost all studies, kinematic sensors are used, either to estimate movements directly or to stabilize other inputs. However, the additional instrumentation of residual body parts seems less practical.

Together with the derived “control strategy landscaping”, our in-depth evaluation of the novel sensors for environmental awareness can serve as decision guidance for future research in this field. New high-frequency RADAR sensors may be the best choice for upcoming object or terrain prediction approaches, perhaps even for event detection strategies. Thanks to their ability to “look through” clothing or cosmetic covers, these sensors could be embedded directly into a prosthetic device, resulting in numerous new possibilities. Before that, however, research must prove that they are sufficiently accurate and efficient.

## Data Availability

Not applicable.

## Abbreviations

2D: Two-dimensional
3D: Three-dimensional
IES: Implicit environmental sensing
EES: Explicit environmental sensing
TOF: Time of fight
LIDAR: Light detection and ranging
RADAR: Radio direction and ranging
IMU: Inertial measurement unit
EMG: Electromyography
GONIO: Electrogoniometer
RANSAC: Random sample consensus
n/a: Not applicable
